# Dysplastic Changes of Peripheral Blood Cells in COVID-19 Infection

**DOI:** 10.4274/tjh.galenos.2020.2020.0342

**Published:** 2021-02-25

**Authors:** Yunus Murat Akçabelen, Dilek Gürlek Gökçebay, Neşe Yaralı

**Affiliations:** 1 University of Health Sciences Turkey, Ankara City Hospital, Clinic of Pediatric Hematology, Ankara, Turkey

**Keywords:** Coronavirus, Blood smear, Dysplasia

A 16-year-old girl with a history of contact with another person with coronavirus disease-2019 (COVID-19) presented with cough, dyspnea, and anosmia. The severe acute respiratory syndrome coronavirus-2 real-time polymerase chain reaction test was positive. Upon physical examination, bilateral medium coarse rales were noticed. The laboratory workup revealed hemoglobin of 16 g/dL, white blood cell count of 5.1x10^9^/L, platelet count of 158x10^9^/L, and serum C-reactive protein level of 1.2 mg/L. B12 and folic acid tests were not performed because she was evaluated in the emergency department. Peripheral smear showed many giant platelets, vacuolated monocytes, and dysplastic neutrophils. We also observed similar dysplastic changes in other children with COVID-19 (Figure 1; May-Grunewald-Giemsa stain; 100^x^).

Dysplastic morphology of blood cells can be noted in both myelodysplastic syndrome and in many nonclonal diseases such as infections, autoimmune disorders, nutritional deficiencies, drugs, or cases of toxins [1]. Prominent morphological abnormalities of the neutrophils and platelets are reported in adult patients with COVID-19 [2,3]. In patients with COVID-19 infection, the upregulation of proinflammatory cytokines was reported [4]. Inhibitory effects of cytokines from virus-infected cells on hematopoiesis may also be responsible for myelodysplastic changes [1].

## Figures and Tables

**Figure 1:**
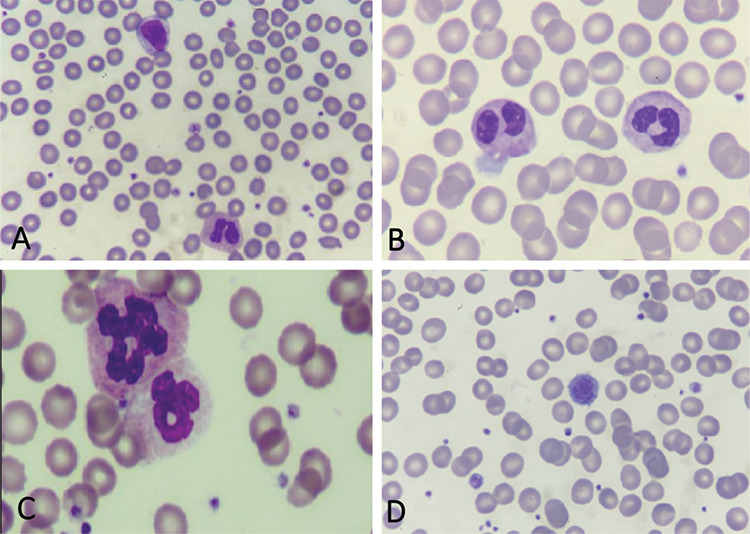
Dysplastic changes in peripheral blood (May-Grunewald-Giemsa stain; 100^x^). **A)** A reactive lymphocyte and pseudo-Pelger-Huet anomaly of the neutrophil. **B)** Pseudo-Pelger-Huet anomaly of neutrophils. **C)** Lobulation anomaly of the neutrophils. **D)** A giant platelet.
